# High-Risk Benign Breast Lesions: An Ontario Health (Cancer Care Ontario) Recommendations Report

**DOI:** 10.3390/curroncol33020067

**Published:** 2026-01-23

**Authors:** Andrea Eisen, Anita Bane, Petrina Causer, Erin Cordeiro, Samantha Fienberg, Anat Kornecki, Ameya Kulkarni, Nicole Look Hong, Talia Mancuso, Derek Muradali, Sharon Nofech-Mozes, Amanda Roberts, Rola Shaheen, Sarah Courtney, Rachael Grove, Muriel Brackstone

**Affiliations:** 1 Division of Medical Oncology, Hamilton Health Sciences, University of Toronto, Toronto, ON M5S 1A1, Canada; eisena@hhsc.ca; 2 Pathology, University Health Network, University of Toronto, Toronto, ON M5G 2C4, Canada; anita.bane@uhn.ca; 3 Radiology, North York General Hospital, University of Toronto, Toronto, ON M2K 1E1, Canada; petrina.causer@nygh.on.ca; 4 Surgical Oncology, The Ottaway Hospital, University of Ottawa, Ottawa, ON K1N 6N5, Canada; ecordeiro@toh.ca; 5 Radiology, Lakeridge Health, University of Toronto, Toronto, ON M5S 1A1, Canada; samantha.fienberg@ontariohealth.ca; 6 Radiology, St. Joseph Health Centre, University of Western Ontario, London, ON N6A 3K7, Canada; anat.kornecki@sjhc.london.on.ca; 7 Radiology, Hamilton Health Sciences Centre, McMaster University, Hamilton, ON L8S 4L8, Canada; kulkarniam@hhsc.ca; 8 Surgical Oncology, Sunnybrook Health Sciences Centre, University of Toronto, Toronto, ON M4N 3M5, Canadaamanda.roberts@sunnybrook.ca (A.R.); 9 Genetic Counselling, Sunnybrook Health Sciences Centre, Toronto, ON M4N 3M5, Canada; talia.mancuso@sunnybrook.ca; 10 Radiology, St. Michael’s Hospital, University of Toronto, Toronto, ON M5G 2C4, Canada; derek.muradali@unityhealth.to; 11 Pathology, Sunnybrook Health Sciences Centre, University of Toronto, Toronto, ON M4N 3M5, Canada; sharon.nofech-mozes@sunnybrook.ca; 12 Radiology, Peterborough Regional Health Centre, Peterborough, ON K9J 7C6, Canada; rola.shaheen@sympatico.ca; 13 Ontario Health (Cancer Care Ontario), 500-525 University Avenue, Toronto, ON M5G 2L3, Canada; sarah.courtney@ontariohealth.ca (S.C.); rachael.grove@ontariohealth.ca (R.G.); 14 Surgical Oncology, University of Western Ontario, London, ON N6A 3K7, Canada

**Keywords:** high-risk, breast cancer, breast imaging, breast biopsy, surgery, clinical guidelines, benign breast lesions

## Abstract

High-risk benign breast lesions are histological abnormalities that present in breast tissue, typically identified by screening or diagnostic imaging. The presence of invasive or in situ breast cancer can be confirmed or ruled out within these lesions, and the risk of developing breast cancer can be reduced by their appropriate management. These potential high-risk lesions reviewed include atypical ductal hyperplasia, mucocele-like lesions, papillary lesions with or without atypia, radial scar/complex sclerosing lesion with or without atypia, atypical lobular hyperplasia, classical lobular carcinoma in situ, pleomorphic/florid lobular carcinoma in situ, flat epithelial atypia, columnar cell change, fibroepithelial lesions with stromal cellularity, spindle cell lesions/mesenchymal lesions, and microglandular adenosis. The lack of a clear consensus on the management of many of these lesions led the Ontario Health (Cancer Care Ontario) (OH-CCO) Breast Cancer Pathway Map Working Group and Breast Cancer Advisory Committee to identify the need for a recommendation document. A multidisciplinary working group was formed, with members representing surgical oncology, radiology, pathology, medical oncology, and genetic counselling. The working group developed a list of high-risk benign lesions to be included in this recommendation report. An updated literature review was completed, and these publications were reviewed by the working group, and recommendations were drafted. When evidence was lacking, the expert opinion was included. These draft recommendations were subjected to an extensive review by experts both within Cancer Care Ontario and across Canada. The recommendations included in this report are relevant to clinicians, primary care physicians, oncologists, radiologists, and pathologists who treat breast cancer and manage breast conditions.

## Simple Summary

High-risk benign breast lesions are abnormalities found in breast tissue that are associated with an increased risk of breast cancer. A working group of specialists, including surgeons, pathologists, and radiologists, who treat breast cancer was developed, and relevant publications were reviewed. Recommendations were then developed for each high-risk benign breast lesion based on this evidence as well as working group expert opinions. This document was reviewed by relevant experts across Canada and is relevant to all healthcare providers with an interest in breast cancer and cancer prevention.

## 1. Introduction

High-risk benign breast lesions are histologic abnormalities that are associated with an increased risk of breast cancer [1]. The appropriate management of such lesions can help to reduce the breast cancer incidence by preventing the progression to invasive disease and/or identifying situations where concurrent in situ or invasive cancer already exists [1]. However, the lack of consensus regarding the management of such lesions on a biopsy has led to variations in the care of these patients [2]. These lesions may present clinically or on screening mammograms. Average-risk breast screening in Canada involves a biennial mammogram in women aged 40–74.

The need for clear management recommendations for high-risk benign breast lesions specific to the Ontario context was identified by the Ontario Health [Cancer Care Ontario] [OH-CCO] Breast Cancer Pathway Map Working Group and Breast Cancer Advisory Committee. A multidisciplinary working group was formed to develop recommendations for the management of high-risk benign breast lesions in Ontario. Working group members represented healthcare professionals who care for patients with breast lesions, including radiology, pathology, surgical oncology, medical oncology, and genetic counselling.

A systematic review of the evidence on management and follow-up strategies for high-risk breast lesions was conducted in 2018, and an updated literature review was conducted in 2022. The most recently published guidance documents were then identified and reviewed [3,4,5]. The working group developed recommendations based on the collated evidence as well as working group members’ expertise. The document was reviewed by the working group, OH-CCO disease site groups [e.g., the Ontario Breast Cancer Advisory Committee], relevant programme leadership and representatives, and additional clinical experts from across Ontario and Canada.

The importance of assessing radiologic–pathologic concordance in the management of high-risk benign breast lesions was discussed and reviewed for each high-risk lesion [6], and the surgical excision of benign high-risk lesions was recommended when there was radiologic–pathologic discordance or when the risk of an upgrade to a malignant diagnosis was considered too high to follow through with conservative imaging. Specifically, for lesions deemed discordant (where the lesion on imaging was concerning but the pathology was benign) and when a risk of upstaging remains, surgical excision is recommended. In cases where the lesion was expected to be benign but a small incidental high-risk lesion was identified, these cases may be treated conservatively depending on the lesion as per the target lesion recommendation sections below. The topic of endocrine prevention [also termed chemoprevention] with tamoxifen, low-dose tamoxifen, or aromatase inhibitors in patients at an elevated risk of developing breast cancer falls outside the scope of this report. However, the authors recommend that these patients should be referred to the appropriate specialist based on institutional practices to receive a tailored discussion of endocrine prevention [7,8]. In Canada, patients are firstly referred to a general surgeon for consultation regarding any concerning breast lesion, typically through a breast diagnostic assessment clinic. A multidisciplinary discussion with a relevant pathologist, radiologist, and/or oncologist is typically recommended as needed for treatment decision-making, prior to or following surgical consultation depending on local institutional referral practice patterns.

The upgrade rate [also known as upstaging] represents the likelihood that the lesion is under-sampled at biopsy and actually contains an in situ or invasive cancer. The upgrade rates presented in this report represent ranges found in the literature, rather than pooled averages. The ‘Resource Guide: Surgical Management of Benign or High-Risk Lesions’ from the American Society of Breast Surgeons [ASBrS] discusses many of the high-risk benign breast lesions included in this report [3]. The ASBrS guide uses a different methodology, which presents pooled meta-analysis percentages. As a result, many of the upgrade rates presented in the ASBrS guide will be lower than those presented in this report.

In conducting this systematic review, three electronic databases and relevant clinical society websites were searched (Ovid Medline, Ovid Embase, and EBSCO CINAHL) in January 2018 and updated in 2022. Searches were limited to the English language, humans, and articles published from 2012 onward. Database-specific search strategies were developed using a combination of the following search terms with Boolean operators (‘and’ and ‘or’): breast lesions of interest, follow-up interventions, and management. Articles were included if they described (1) follow-up interventions for the effective management of high-risk breast lesions and/or probably benign lesions or (2) the risk of upstaging of high-risk lesions. The initial search strategy identified 4678 publications, of which 160 were confirmed relevant articles. With regard to calculating the range of the upstage risk specifically, 110 of these studies discussed upstage rates. Most studies had sample sizes of several hundred of patients, and none had a lesion-specific sample size less than 40.

The literature review and the recommendations endorsed by the working group are presented for each of the following high-risk benign breast lesions: atypical ductal hyperplasia [ADH], mucocele-like lesions [MLLs], papillary lesions, radial scars/complex sclerosing lesions, atypical lobular hyperplasia [ALH], lobular carcinoma in situ [LCIS], columnar cell change, flat epithelial atypia, fibroepithelial lesions with increased stromal cellularity, spindle cell lesions/mesenchymal lesions, and microglandular adenosis [MGA]. The full recommendation report is available on the Ontario Health [Cancer Care Ontario] website at https://www.cancercareontario.ca/en/guidelines-advice/types-of-cancer/79886 (accessed on 17 January 2026). A summary of the recommendations is presented in Table 1.

## 2. Recommendations

### 2.1. Atypical Ductal Hyperplasia [ADH]

Upgrade Rate at Excision: 7–52.9% [9,10,11,12,13,14,15,16,17,18,19,20,21,22,23,24,25,26,27,28,29,30,31,32,33,34,35,36,37,38,39,40,41,42,43,44,45,46,47,48,49,50,51,52,53,54,55].

#### 2.1.1. Recommendations

- Excision recommended, refer to a surgeon [although not every case requires excision, see considerations below]. Decision to excise will be made with the patient, considering comorbidities and patient preferences in shared decision-making.
- If excised, proceed to annual screening after excision. Individuals with high-risk lesions should have annual screenings as per OBSP recommendations [see Breast Screening Recommendations Summary|Cancer Care Ontario].
- If after consultation with a surgeon, the decision was made not to excise, follow for 2 years, with follow-up at 6, 12, and 24 months using the imaging modality with which it was originally seen [ordered by the referring physician], then proceed to annual screening.
- Provide breast cancer risk assessment and counselling about breast cancer risk-reducing options [i.e., endocrine prevention, see above].
- Pathologists should specify the extent of ADH in the specimen [focal or incidental vs. present on multiple cores vs. prominent with differential of low-grade ductal carcinoma in situ].

#### 2.1.2. Considerations

Some cases of ADH may not require excision. The factors to be considered for observation are as follows [44,56,57,58,59]:

- Characteristics/distribution of mammographic calcifications [i.e., presence of more benign-appearing microcalcifications without any associated mass/distortion].
- Smaller lesion size on imaging (<1 cm) resulting in complete or near complete removal of calcifications on post-biopsy imaging.
- Larger sample size [i.e., biopsy using vacuum-assisted biopsy where the majority (>50%) of lesion is sampled].
- Small volume of ADH in the core needle biopsy specimen as described by pathologist (i.e., only 1–2 foci of ADH less than 2 mm in one core biopsy specimen and no evidence of cellular necrosis or micropapillary features).
- Absence of additional high-risk lesions [i.e., any other high-risk lesions outlined in this document].

### 2.2. Mucocele-like Lesions [MLLs]

Upgrade Rate at Excision: 0–17% [60,61,62,63,64,65,66,67,68,69].

#### 2.2.1. Recommendations

- Referral to a surgeon for consultation is recommended. Routine excision is not required if pathology is concordant with radiology assessment. Excision should be discussed in context of patient preference and radiologic risk factors.
- Risk of upstaging to ductal carcinoma in situ (DCIS) or mucinous carcinoma is higher when the biopsy shows atypia, specifically ADH [61]; therefore, MLLs with atypia should undergo excision, and those without atypia do not routinely require excision.
- If excised, proceed to annual screening after excision. Individuals with high-risk lesions should have annual screening as per OBSP recommendations [see Breast Screening Recommendations Summary|Cancer Care Ontario].
- If unexcised, follow for 2 years, with follow-up at 6, 12, and 24 months using the imaging modality with which it was originally seen [ordered by the referring physician], then proceed to annual screening.
- Pathologists should specify whether the lesion was examined on additional levels.

#### 2.2.2. Considerations

Excision recommended for MLLs with atypia as the upgrade rate to breast cancer is as high as 31% [61,63,69].

### 2.3. Pure Papillary Lesions with Atypia

Upgrade Rate at Excision [pure papillary lesions with atypia]: 18.7–56.5% [70,71,72,73,74].

#### Recommendations

- Excision recommended, refer to a surgeon. Decision to excise will be made with the patient, considering comorbidities and patient preference in shared decision-making.
- If excised, proceed to annual screening after excision. Individuals with high-risk lesions should have annual screening as per OBSP recommendations [see Breast Screening Recommendations Summary|Cancer Care Ontario].
- If after consultation with a surgeon, the decision was made not to excise, follow for 2 years, with follow-up at 6, 12, and 24 months using the imaging modality with which it was originally seen [ordered by the referring physician], then proceed to annual screening.
- Provide breast cancer risk assessment and counselling about breast cancer risk-reducing options [i.e., endocrine prevention].
- Pathologists should comment on the extent of atypia [i.e., papilloma involved by ADH, at least ADH, as opposed to the entire lesion being atypical].

### 2.4. Pure Papillary Lesions Without Atypia

Upgrade Rate at Excision [pure papillary lesions without atypia]: 0–14.3% [70,71,72,73,74].

#### 2.4.1. Recommendations

- Referral to a surgeon for consultation is recommended. Routine excision is not required if pathology is concordant with radiology assessment. Excision should be discussed in context of patient preference and radiologic risk factors.
- If excised [i.e., based on patient preference or imaging discordance], proceed to annual screening after excision.
- If unexcised and no growth, follow for 2 years, with follow-up at 12 and 24 months using the imaging modality with which it was originally seen [ordered by the referring physician], then proceed to average-risk screening.
- Pathologically incidental papillary lesions without atypia, defined as small papillary lesions seen on biopsy away from the targeted radiologic abnormality, do not require follow-up. Only the index lesion that prompted the biopsy requires follow-up according to the recommendations for that lesion.

#### 2.4.2. Considerations

- Some papillary lesions without atypia may be considered for excision. This shared decision-making between surgeon and patient will consider patient preference. The factors to be weighed in the consideration of excision include the following:
  (1) >54 years of age [75];
  (2) Lesions >1 cm at largest diameter [75,76];
  (3) Lesions with dynamic change in size [>5 mm growth in max diameter in 6 months] [4];
  (4) Associated ipsilateral breast cancer [consensus];
  (5) If presence of associated high-risk lesion on excision will alter management [e.g., offer chemoprevention] [7];
  (6) Radiologic–pathologic discordance [76];
  (7) Symptoms interfering with perceived quality of life [e.g., bloody nipple discharge, palpable mass] [3,4,77].

The American Society of Breast Surgeons recommends surgical excision for symptomatic papillary lesions irrespective of atypia [3]. For these asymptomatic lesions <1 cm with adequate sampling [i.e., vacuum-assisted biopsy or extensive sampling of lesion] and radiologic–pathologic concordance, a 6-month imaging follow-up rather than referral can be considered. This should be decided based on institutional guidelines.

### 2.5. Pure Radial Scars/Complex Sclerosing Lesions

Upgrade Rate at Excision [all pure radial scars/complex sclerosing lesions]: 0–25.0% [12,15,18,22,24,28,29,43,51,78,79,80,81,82,83,84,85,86,87,88,89,90,91,92,93,94,95,96,97,98,99,100,101,102,103,104].

#### 2.5.1. Pure Radial Scars/Complex Sclerosing Lesions with Atypia

##### Recommendations

- Excision recommended, refer to a surgeon. If atypia is present, the rate of upgrade is as high as 35% [51], and therefore excision of radial scar/complex sclerosing lesion with atypia is recommended. Decision to excise will be made with the patient, considering comorbidities and patient preference in shared decision-making.
- If excised, proceed to annual screening after excision. Individuals with confirmed high-risk lesions should have annual screening as per OBSP recommendations [see Breast Screening Recommendations Summary|Cancer Care Ontario].
- If after consultation with a surgeon, the decision was made not to excise, follow for 2 years, with follow-up at 6, 12, and 24 months using the imaging modality with which it was originally seen [ordered by the referring physician], then proceed to annual screening.
- Provide breast cancer risk assessment and counselling about breast cancer risk-reducing options.

#### 2.5.2. Pure Radial Scars/Complex Sclerosing Lesions Without Atypia

##### Recommendations

- Referral to a surgeon for consultation is recommended. Routine excision is not required if pathology is benign concordant with radiology assessment. Excision should be discussed in context of patient preference and radiologic risk factors.
- If excised, proceed to average-risk screening after excision.
- If unexcised and no growth, follow for 2 years, with follow-up at 12 and 24 months using the imaging modality with which it was originally seen [ordered by the referring physician], then proceed to average-risk screening. Follow-up at 6 months can be considered according to institutional practice.
- Pathologically and radiologically incidental radial scars/complex sclerosing lesions without atypia reported in the biopsy sample, performed to diagnose a separate targeted radiologic abnormality, do not require follow-up since these lesions are <5 mm and concordant with benign imaging. Only the targeted index lesion requires follow-up according to the recommendations for that lesion.

##### Considerations

- Some pure radial scars/complex sclerosing lesions without atypia may require excision. The factors to be considered for excision include the following:
  (1) Family history of breast or ovarian cancer [105,106];
  (2) Associated with a mass [107,108];
  (3) Radiologic–pathologic discordance [107,109], where the radiologist is concerned about upstaging of the lesion but pathology does not demonstrate any atypia.

Incidental radial scars/complex sclerosing lesions without atypia should be managed as if they were the index lesion, with referral to a surgeon for consultation, unless the radial scar/complex sclerosing lesion is very small in size [< 5 mm and concordant].

### 2.6. Atypical Lobular Hyperplasia [ALH]

Upgrade Rate at Excision: 1.7–36% [12,14,15,18,21,22,24,25,28,29,30,32,33,43,78,80,81,88,94,105,106,107,108,109,110,111,112,113,114,115,116,117,118,119,120,121,122,123,124,125,126,127,128,129,130].

#### 2.6.1. Recommendations

- Referral to a surgeon for consultation is recommended. Generally, ALH does not require excision if breast imaging has ruled out any other lesions and is reported as concordant. Excision should be discussed in context of patient and radiologic risk factors.
- If excised, proceed to annual screening after excision. Individuals with confirmed high-risk lesions should have annual screening as per OBSP recommendations [see Breast Screening Recommendations Summary|Cancer Care Ontario].
- If the imaging-targeted lesion that was biopsied and confirmed ALH was not excised and shows no growth during serial imaging, follow for 2 years, with follow-up at 12 and 24 months using the imaging modality with which it was originally seen [ordered by the referring physician], then proceed to annual screening. Follow-up at 6 months can be considered according to institutional practice.
- Provide breast cancer risk assessment and counselling about breast cancer risk-reducing options (i.e., endocrine prevention).
- Pathologically incidental ALH, defined as lesions noted in an area unrelated to the targeted radiologic abnormality, do not require short-interval follow-up; however, closer surveillance with annual imaging may be considered. Only the targeted index lesion that prompted the biopsy requires specific follow-up according to the recommendations for that lesion.

#### 2.6.2. Considerations

- Referral to a surgeon for consideration of excision is recommended if additional risk factors are present, including the following:
  (1) Family history of breast or ovarian cancer [105,106];
  (2) Associated with a mass [107,108];
  (3) Associated with other high-risk lesions such as ADH and non-classic LCIS [131,132];
  (4) Radiologic–pathologic discordance [107,109,111,131] where the radiologist remains concerned regarding upstaging risk.

Consider bilateral contrast-enhanced imaging for patients with ALH prior to surgical consultation due to the associated increased risk of bilateral malignancy. Repeat the core needle biopsy with vacuum assistance to increase tissue sampling, where available, prior to surgical consultation as an option to increase diagnostic accuracy in cases of discordance.

Radiologists should report the presence of microcalcifications within core biopsy samples at the time of the diagnostic biopsy. Pathologists should likewise report the presence of microcalcifications and whether they are associated with the target lesion or seen in adjacent normal tissue, as this will allow the radiologist to provide an accurate concordance opinion as well as guide management, through consultation between the surgeon and radiologist.

### 2.7. Classic Lobular Carcinoma in Situ [LCIS]

Upgrade Rate at Excision: 1.7–36% [12,15,18,21,22,24,26,28,29,30,33,43,78,80,81,88,94,105,106,107,109,110,111,112,113,114,115,116,117,118,119,120,121,122,123,124,125,126,127,128,129,130,133,134].

#### 2.7.1. Recommendations

- Referral to a surgeon for consultation is recommended. Generally, classic LCIS does not require excision if breast imaging has ruled out any other lesions and is reported as concordant. Excision should be discussed in context of patient and radiologic risk factors.
- The American Society of Breast Surgeons’ ‘*Resource Guide: Surgical Management of Benign or High-Risk Lesions*’ reports an upstage rate of 0–4% in cases where there is concordance between radiologist and pathologist [3].
- If excised for definitive diagnosis, proceed to annual screening after excision. Individuals with high-risk lesions should have annual screening as per OBSP recommendations [see Breast Screening Recommendations Summary|Cancer Care Ontario].
- If unexcised and no growth or change, follow for 2 years, with follow-up at 12 and 24 months using the imaging modality with which it was originally seen [ordered by the referring physician], then proceed to annual screening. Follow-up at 6 months can be considered according to institutional practice.
- Provide breast cancer risk assessment and counselling about breast cancer risk-reducing options [ALH and classic LCIS are non-obligate precursor lesions, implying the presence of either denotes an increased risk of cancer in either breast in the order of 1–1.5% per year] [3].

#### 2.7.2. Considerations

It is not routine practice in Ontario to quantify the amount of classic LCIS in a core biopsy, and it is suggested that educational initiatives should be considered for this guidance document.

Radiologists should report the presence of microcalcifications within core biopsy samples at the time of the diagnostic biopsy. Pathologists should likewise report the presence of microcalcifications and whether they are associated with the target lesion or seen in adjacent normal tissue, as this will allow the radiologist to provide an accurate concordance opinion as well as guide management, through consultation between the surgeon and radiologist.

Referral to a surgeon for the consideration of excision is recommended if additional risk factors are present, including the following:

- Extensive LCIS (based on pathologist assessment, for example describe whether LCIS involves >4 terminal ductal lobular units) [107];
- Associated with other high-risk lesions such as ADH and non-classic LCIS [131,132];
- Radiologic–pathologic discordance [107,111,115,131].

Consider bilateral contrast-enhanced imaging for patients with classic LCIS prior to surgical consultation due to the associated increased risk of bilateral malignancy.

Referral to genetic counselling is recommended for patients with a family history of breast cancer if appropriate based on current provincial guidelines [30].

### 2.8. Variant/Non-Classic Lobular Carcinoma in Situ [Pleomorphic or Florid]

#### 2.8.1. Recommendations

- Excision recommended, refer to a surgeon. Decision to excise will be made with the patient, considering comorbidities and patient preference in shared decision-making.
- If excised, proceed to annual screening after excision. Individuals with high-risk lesions should have annual screening as per OBSP recommendations [see Breast Screening Recommendations Summary|Cancer Care Ontario].
- If there is no growth and, after consultation with a surgeon, the decision was made not to excise, follow for 2 years, with follow-up at 6, 12, and 24 months using the imaging modality with which it was originally seen [ordered by the referring physician], then proceed to annual screening.
- Provide breast cancer risk assessment and counselling about breast cancer risk-reducing options.

#### 2.8.2. Considerations

- Consider bilateral contrast-enhanced imaging for patients with variant/non-classic LCIS prior to surgical consultation due to associated increased risk of bilateral malignancy.

### 2.9. Flat Epithelial Atypia [Columnar Cell Change with Atypia]

Upgrade Rate at Excision: 0–18.3% [9,12,14,15,16,18,21,28,29,31,40,43,45,81,135,136,137,138,139,140,141,142,143,144,145,146,147,148,149,150,151,152,153,154,155].

#### 2.9.1. Recommendations

- Referral to a surgeon for consultation is recommended. Routine excision is not required if pathology is concordant with radiology assessment. Excision should be discussed in context of patient preference and radiologic risk factors.
- If excised, proceed to annual screening after excision. Individuals with high-risk lesions should have annual screening as per OBSP recommendations [see Breast Screening Recommendations Summary|Cancer Care Ontario].
- If unexcised and no growth, follow for 2 years, with follow-up at 12 and 24 months using the imaging modality with which it was originally seen [ordered by the referring physician], then proceed to annual screening. Follow-up at 6 months can be considered according to institutional practice.
- Provide breast cancer risk assessment and counselling about breast cancer risk-reducing options.

#### 2.9.2. Considerations

Referral to a surgeon for consideration of excision is recommended if additional risk factors are present, including the following:

- Presence of ADH or ALH [135,136,138,141,147,156,157];
- Presence of residual calcifications post-core biopsy where concordance is not clear;
- Quality of the core needle excision [138] insufficient for definitive diagnosis;
- Radiologic–pathologic discordance [consensus].

If extensive calcifications are felt to be inadequately sampled [15,135,139,142,143,144,146] such that a concordance with the pathology is not clear, a repeat core biopsy (with vacuum assistance if available) should be performed prior to surgical consultation.

Radiologists should report the presence of microcalcifications within core biopsy samples at the time of the diagnostic biopsy. Pathologists should likewise report the presence of microcalcifications and whether they are associated with the target lesion or seen in adjacent normal tissue, as this will allow the radiologist to provide an accurate concordance opinion as well as guide management, through consultation between the surgeon and radiologist.

Referral to genetic counselling can be considered, if appropriate, in patients with a family history of breast cancer [140].

### 2.10. Columnar Cell Change Without Atypia

#### 2.10.1. Recommendations

- Follow-up at 12 months using the imaging modality with which it was originally seen [ordered by the referring physician]; then proceed to average-risk screening. Follow-up at 6 months can be considered according to institutional practice.

#### 2.10.2. Considerations

- The opinion of the working group is that this lesion is benign. A 12-month follow-up study should be considered if the subject is not receiving annual screening. Follow-up should only be considered if there is discordance with pathology and imaging.
- Columnar cell change without atypia is NOT a high-risk lesion but is included in this document to clarify that this common finding in fibrocystic breast change does not require short-interval follow-up imaging or surgical consultation.

### 2.11. Fibroepithelial Lesions with Increased Stromal Cellularity

Upgrade Rate at Excision: 7.7–29% [158,159,160,161,162,163,164,165].

#### 2.11.1. Recommendations

- Referral to a surgeon for consultation is recommended. Routine excision is not required if pathology is concordant with radiology assessment. Excision should be discussed in context of patient preference and radiologic risk factors.
- If excised, proceed to average-risk screening after excision.
- If unexcised and no growth, follow for 2 years, with follow-up at 12 and 24 months using the imaging modality with which it was originally seen [ordered by the referring physician], then proceed to average-risk screening. Follow-up at 6 months can be considered according to institutional practice.

#### 2.11.2. Considerations

The following factors may be considered when evaluating for excision; however, the working group did not reach a consensus on which of the following would support a recommendation of excision:

- Heterogeneous echotexture, lack of internal vascularity, and high BIRADS^®^ score [≥4b] [159];
- Fibroepithelial lesions where phyllodes tumours cannot be ruled out on pathology [given that phyllodes and fibroadenoma cannot always be resolved confidently on the needle core specimen alone] [158];
- Patients with lesions >2 cm that are potentially new or growing [consensus];
- Radiologic–pathologic discordance [consensus].

### 2.12. Spindle Cell Lesions/Mesenchymal Lesions

Spindle cell lesions of the breast include both benign and malignant diagnoses that may be challenging to classify [166]. The management of the benign lesions depends on the pathologic subclassification.

#### 2.12.1. Recommendations

- Obtain pathology review of core biopsy by expert breast or soft tissue pathologist with capacity to perform appropriate immunohistochemical analysis.
- Excision recommended, refer to a breast surgeon. Decision to excise will be made with the patient, considering comorbidities and patient preference in shared decision-making.
- If excised, proceed to annual screening or follow-up according to final diagnosis [determined by surgeon, depending on diagnosis of soft tissue sarcoma versus benign mesenchymal lesion].

#### 2.12.2. Considerations

These cases require an expert pathology review and the involvement of a specialized breast surgeon.

### 2.13. Microglandular Adenosis [MGA]

MGA is a rare benign lesion that mimics invasive triple-negative breast cancer [167]. The working group felt that, although rare, it was important to include this lesion because of the potential for misclassification.

#### Recommendations

- Obtain pathology review of core biopsy by expert breast pathologist.
- Excision recommended if there is any question as to whether the lesion harbours malignancy; refer to a breast surgeon. Decision to excise will be made with the patient, considering comorbidities and patient preference in shared decision-making.
- If excised, proceed to average-risk screening or follow-up according to final diagnosis.
- Pathologists need to clarify whether MGA is incidental or predominant lesion. Conservative management can be considered in former. Radiologic–pathologic correlation should help drive the management.
- Pathologists should report any form of atypia, either cytologic, architectural, or mitotic activity.
- Excision is recommended for atypical MGA due to its association with triple-negative breast carcinoma.

## 3. Conclusions

High-risk benign breast lesions are challenging because of the need to determine a concordance between the radiologist and pathologist for each lesion as well as incorporating the individual breast density/enhancement, patient-specific breast cancer risk, and patient-informed treatment preferences. For lesions that are considered inadequately sampled by a core needle biopsy or for which there remains radiologic/pathologic discordance, a repeat vacuum-assisted biopsy can result in the sampling of the majority of the lesion when it is smaller in size, avoiding surgical excision depending on recommendations specific to the lesion. Currently, Ontario healthcare does not fund vacuum-assisted excisions as an alternative to surgery, but for institutions where this option exists, this can be an option to remove the lesion when the likelihood of malignancy is low.

The lack of standardized approaches to these lesions along with significant improvements in accuracy in imaging modalities over time have resulted in a wide variety of practices in diagnostic assessment and treatment for these patients. This confusion can create additional stress for patients and healthcare providers alike.

It is hoped that this recommendation report will provide some guidance for the standardized care of high-risk lesions in the Canadian healthcare system and that it will be helpful to providers, including surgeons, radiologists, pathologists, medical oncologists, and genetic counsellors.

## Figures and Tables

**Table 1:** Summary of high-risk benign breast lesion management recommendations.

Lesion	Initial Management	Follow-Up
Atypical Ductal Hyperplasia	Excision recommended, refer to a surgeon. Provide breast cancer risk assessment and counselling about breast cancer risk reduction options.	If excised, return to annual screening after excision. If unexcised, follow for 2 years at 6, 12, and 24 months using the imaging modality with which it was originally seen, then annual screening.
Mucocele-Like Lesions	Referral to a surgeon for consultation is recommended. Routine excision is not required if pathology is concordant with radiology assessment. Excision should be discussed in context of patient preference.	If excised, return to annual screening after excision. If unexcised, follow for 2 years at 6, 12, and 24 months using the imaging modality with which it was originally seen, then annual screening.
Pure Papillary Lesions with Atypia	Excision recommended, refer to a surgeon. Provide breast cancer risk assessment and counselling about breast cancer risk reduction options.	If excised, return to annual screening after excision. If unexcised, follow for 2 years at 6, 12, and 24 months using the imaging modality with which it was originally seen, then annual screening.
Pure Papillary Lesions without Atypia	Referral to a surgeon for consultation is recommended. Routine excision is not required if pathology is concordant with radiology assessment. Excision should be discussed in context of patient preference.	If excised, return to annual screening after excision. If unexcised and no growth, follow for 2 years, with follow-up at 12 and 24 months using the imaging modality with which it was originally seen, then average-risk screening.
Pure Radial Scars/Complex Sclerosing Lesions with Atypia	Excision recommended, refer to a surgeon. Provide breast cancer risk assessment.	If excised, return to annual screening after excision. If unexcised, follow for 2 years, with follow-up at 6, 12 and 24 months using the imaging modality with which it, was originally seen, then annual screening.
Pure Radial Scars/Complex Sclerosing Lesions without Atypia	Referral to a surgeon for consultation is recommended. Routine excision is not required if pathology is concordant with radiology assessment. Excision should be discussed in context of patient preference.	If excised, return to screening after excision. If unexcised and no growth, follow for 2 years, with follow-up at 12 and 24 months using the imaging modality with which it was originally seen, then average-risk screening.
Atypical Lobular Hyperplasia	Consultation recommended, refer to a surgeon. Provide breast cancer risk assessment and counselling about breast cancer risk reduction options.	If excised, return to annual screening after excision. If unexcised and no growth, follow for 2 years, with follow-up at 12 and 24 months using the imaging modality with which it was originally seen, then annual screening.
Classic Lobular Carcinoma in Situ	Consultation recommended, refer to a surgeon. Provide breast cancer risk assessment and counselling about breast cancer risk reduction options.	If excised, return to annual screening after excision. If unexcised and no growth, follow for 2 years, with follow-up at 12 and 24 months using the imaging modality with which it was originally seen, then annual screening.
Variant/Non-Classic Lobular Carcinoma in Situ	Excision recommended, refer to a surgeon. Provide breast cancer risk assessment and counselling about breast cancer risk reduction options.	If excised, return to annual screening after excision. If unexcised, follow for 2 years, with follow-up at 6, 12, and 24 months using the imaging modality with which it was originally seen, then annual screening.
Flat Epithelial Atypia	Referral to a surgeon for consultation is recommended. Routine excision is not required if pathology is concordant with radiology assessment. Excision should be discussed in context of patient preference.	If excised, return to annual screening after excision. If unexcised and no growth, follow for 2 years, with follow-up at 12 and 24 months using the imaging modality with which it was originally seen, then annual screening.
Columnar Cell Change without Atypia	No consultation is required if imaging concordant.	follow-up at 12 months using the imaging modality with which it was originally seen, then resume average-risk screening.
Fibroepithelial Lesions with Increased Stromal Cellularity	Referral to a surgeon for consultation is recommended. Routine excision is not required if pathology is concordant with radiology assessment. Excision should be discussed in context of patient preference.	If excised, return to screening after excision. If unexcised and no growth, follow for 2 years, with follow-up at 12 and 24 months using the imaging modality with which it was originally seen, then resume average-risk screening.
Spindle Cell Lesions/Mesenchymal Lesions	These cases require expert pathology review and involvement of a specialized breast surgeon. Excision recommended, refer to a surgeon.	If excised, return to screening or follow-up according to final diagnosis.
Microglandular Adenosis	Obtain pathology review of core biopsy by expert breast pathologist. Excision recommended, refer to a surgeon.	If excised, return to screening or follow-up according to final diagnosis.

## Data Availability

Data used in this document is derived from public domain sources (see all references listed below).

## Abbreviations

The following abbreviations are used in this manuscript:

ADH	Atypical Ductal Hyperplasia
MLL	Mucocele-Like Lesion
OBSP	Ontario Breast Screening Program
ALH	Atypical Lobular Hyperplasia
LCIS	Lobular Carcinoma In Situ
MGA	Microglandular Adenosis
BiRADS	Breast Imaging Reporting and Data System
